# Seafood During Pregnancy and Lactation and Child Neurocognitive Development: A Systematic Review

**DOI:** 10.1016/j.advnut.2025.100414

**Published:** 2025-04-25

**Authors:** Lauren E O’Connor, Maureen K Spill, Sanjoy Saha, Arin A Balalian, Julie S Davis, Amanda J MacFarlane

**Affiliations:** Texas A&M Agriculture, Food and Nutrition Evidence Center, Fort Worth, Texas, United States

**Keywords:** fish, shellfish, omega-3 fatty acids, childhood, adolescence, infants and toddlers

## Abstract

**Background:**

Assessing seafood as a food group, rather than as a source of omega-3 fatty acids or contaminants, may better inform dietary guidance for pregnancy and lactation.

**Objectives:**

This study aims to assess relationships between seafood consumption during pregnancy and lactation and neurocognitive development in the child.

**Methods:**

Three electronic databases were searched up to September 2024 to update a previous search from 2000 to 2019. Articles were included if seafood intake during pregnancy or lactation and a child outcome was assessed [neurocognitive development including cognitive, social–emotional, behavioral, movement/physical, language/communication, and aggregate scores as well as depression, anxiety, attention-deficit/hyperactivity disorder (ADHD), and autism spectrum disorder (ASD)]. Articles were screened at title, abstract, and full-text levels independently by 2 analysts. Data were extracted, quality checked, and synthesized narratively considering the direction, magnitude, and statistical significance of results. The risk of bias was assessed using study design-specific tools. Certainty of evidence was assessed using Grading of Recommendations Assessment, Development and Evaluations.

**Results:**

Forty articles [1 randomized controlled trial (RCT), 24 prospective cohorts, and 1 retrospective cohort] during pregnancy were identified; none for lactation. Evidence suggested relationships between higher seafood consumption and better social–emotional and behavioral development in children and adolescents aged 0–18 y as well as better aggregate scores of development for those <4 y. The certainty of the evidence was very low to moderate due to the lack of RCTs. Evidence for overall cognitive development was inconsistent but higher seafood may be related to better attention, reasoning and problem-solving, and verbal intelligence. However, evidence was limited in the number of studies and ages assessed. Evidence was inconsistent for movement/physical and language/communication development, and a paucity of studies was found for ADHD and ASD.

**Conclusions:**

Seafood consumption within currently recommended amounts during pregnancy may be associated with better social–emotional, behavioral, and aggregate scores of development in the child, as well as potentially some aspects of cognitive development.

This study was registered at PROSPERO as CRD42023432844.

**Statement of significance**

Available evidence suggested that there was a relationship between seafood intake during pregnancy and better social–emotional, behavioral, and aggregate development outcomes in the child.

## Introduction

Historical dietary recommendations for pregnancy and lactation focused primarily on limiting seafood to minimize fetal methylmercury exposure [1]. This is because high exposure to methylmercury can be harmful to a fetus or infant and have adverse impacts on various developmental outcomes [2]. However, the effects of methylmercury exposure from seafood specifically remain unclear [3] due to the potential beneficial effects of seafood consumption for child neurocognitive development [4]. For example, higher blood methylmercury concentrations of pregnant people from countries that eat 10 times the amount of seafood as people in the United States were not associated with delayed neurocognitive development in children aged 9 mo–9 y [[5], [6], [7]]. There is also research to suggest that higher seafood intake during pregnancy and lactation may be associated with positive neurocognitive development in the child [4]. Thus, in more recent dietary guidance, pregnant and lactating people are encouraged to consume 8–12 ounces/wk of seafood, while still prioritizing low-mercury options [8]. This shift in recommendation is in part attributed to higher intakes of long-chain PUFA (i.e. DHA and eicosatetraenoic acid) and other essential vitamins and minerals from seafood [9]. Focusing on seafood as a food group rather than as a vehicle for specific nutrients or contaminants is a comprehensive approach to inform dietary recommendations for pregnancy and lactation to improve child neurocognitive development [9].

In 2022, the National Academies of Sciences, Engineering, and Medicine (NASEM) convened an expert committee called The Role of Seafood in Child Growth and Development to review the nutrition and toxicological evidence about seafood intake and child development [10]. This committee was sponsored by the United States Food and Drug Administration, Environmental Protection Agency, Department of Agriculture, and National Oceanic and Atmospheric Administration to inform future federal guidance on seafood intake. The committee approached this task by evaluating consumption trends and barriers as well as commissioning a suite of systematic reviews that either addressed *de novo* research questions or updated previous systematic reviews conducted by the 2020 Dietary Guidelines Advisory Committee (DGAC) [11, 12]. As part of this series of systematic reviews conducted for the NASEM committee, we provide an updated assessment of relationships between seafood consumption during pregnancy and lactation and neurocognitive development in the child.

## Methods

Our systematic review was an update of the 2020 DGAC systematic review [11]. As tasked by the NASEM committee [10], we replicated the 2020 DGAC literature search strategy with an updated date range to identify eligible studies that have been published since the 2020 DGAC review. We completed data extraction, risk-of-bias assessments, and data synthesis on all of the included studies identified from the updated literature search as well as those included in the 2020 DGAC review. We did this to ensure that the same methods, including an updated risk-of-bias assessment tool, were applied to all studies under consideration. As required by the NASEM committee [10], our protocol reflected the 2020 DGAC protocol, but with the updated search dates, and was preregistered on PROSPERO (CRD42023432844) before the conduct of the literature review. The protocol included the review questions, general search strategy, inclusion/exclusion criteria, risk-of-bias assessment, and synthesis plan including heterogeneity investigation. Our reporting for this manuscript adhered to the PRISMA guidelines [13] (Supplemental Appendix 1) and our methodology met the criteria to be considered a high-quality systematic review according to the A MeaSurement Tool to Assess systematic Reviews (AMSTAR) 2 critical appraisal tool [14] (Supplemental Appendix 2).

The Population, Intervention/Exposure, Comparator, Outcome (PI/ECO) framework is described in Supplemental Figure 1. In brief, we included randomized controlled trials (RCTs), prospective cohort studies (PCSs), and retrospective cohort studies that compared different types, amounts, sources, frequency, or timing of seafood consumption during pregnancy or lactation and neurocognitive development outcomes in the children aged 0–18 y. These study designs were included to reflect the DGAC protocol. The eligible neurocognitive development outcomes also were informed by the DGAC protocol and included cognitive development, social–emotional and behavioral development, movement/physical development, language/communication development, depression, anxiety, attention-deficit/hyperactivity disorder (ADHD), and autism spectrum disorder (ASD) (Supplemental Table 1).

### Search strategy

This systematic review included articles identified in the previous DGAC review, from which studies were searched from January 2000 to October 2019. For this update, our search identified additional articles published from October 2019 until 6 September, 2024. The full search strategy is shown in Supplemental Table 2.

### Screening

Screening followed a similar methodology used for the DGAC review [12] which included title, abstract, and full-text screening completed by 2 independent analysts at each level. All levels of screening were conducted in DistillerSR (DistillerSR, Evidence Partners; 2020). A pilot was completed on ≥25 articles to ensure screening forms were adequate and that analysts interpreted the eligibility criteria similarly. The list of inclusion and exclusion criteria used for screening is shown in Supplemental Table 3. Title screening was used to exclude clearly irrelevant studies; any disagreements automatically moved to the next level. Any disagreements on whether to include or exclude an article at the abstract or full-text level were discussed and resolved by the 2 analysts. If necessary, a third party was consulted to resolve differences. Manual citation searching was conducted by reviewing the reference lists of all included articles.

### Data extraction

Data from all articles were extracted by a trained analysts using a systematic approach and a standardized data extraction form. A second analyst reviewed all extracted data for accuracy and completeness. Any suggested changes were discussed between the analysts. If necessary, a third analyst was consulted. The following data were extracted, as available, from each article: study characteristics including author name, publication year, study design, study name, country, baseline sample size, and funding source; participant characteristics including mother’s age, child sex (% female), race/ethnicity, socioeconomic status, maternal anthropometrics, gestational weight gain, and infant feeding practices; intervention/exposure details including definition/description, assessment method, seafood intake amount and type, child levels of nutrients from seafood including omega-3 polyunsaturated fatty acids, iodine, selenium, iron, fish protein, and vitamin D, and maternal/infant levels of mercury; confounders including key confounders accounted for, key confounders not accounted for, and other confounders accounted for as described in Supplemental Figure 1; outcome(s) and results including outcome subcategories (described in Supplemental Table 1), outcome assessment tool, outcome assessment methods including subscale, child age at outcome assessment, results, analytical sample size, study limitations, summary of results, and quantified data as needed for synthesis. Data were extracted as reported; if data were unclear or missing, then it is noted throughout the manuscript. The authors were not contacted for missing data.

### Risk of bias

Risk of bias was assessed for all included studies independently by 2 analysts using 1 of the following tools depending on study design: Version 2 of the Cochrane risk-of-bias tool for randomized trials (ROB 2.0) [15], the Risk Of Bias In Non-randomised Studies - of Interventions (ROBINS-I) [16], or the Risk Of Bias In Non-randomized Studies - of Exposure (ROBINS-E) [17]. The analysts piloted the tools on 2–3 articles to ensure a consistent approach and interpretation. Further, on completion of the dual, independent risk-of-bias assessments, domain-level ratings were compared between the 2 reviewers. Discrepancies were resolved through discussion and if necessary, a third reviewer was consulted. The overall rating was equivalent to the highest risk of bias rating across all domains.

### Data synthesis

Meta-analyses were planned, as indicated in the preregistered protocol, but not performed due to variations in the outcome assessment tools, scoring systems and algorithms used, types of relationships assessed (e.g. continuous, dichotomous), types of statistical analyses performed (e.g. odds ratio, trends across quintiles), and reported estimands. Therefore, results were narratively synthesized and sources of heterogeneity were also explored narratively, as described next, instead of using statistical tests. This was deemed a necessary deviation from the protocol based on the limitations of the evidence included.

Important sources of heterogeneity were population characteristics, seafood type, and suboutcomes assessed within each developmental domain. To adequately investigate these potential sources of heterogeneity, we first organized the specific outcome assessments within each outcome into suboutcomes (e.g. processing speed and attention were suboutcomes of cognitive development). Several resources were used to inform this organization, which are described in detail in Supplemental Table 1. Due to the breadth of suboutcomes reported in the studies, this resulted in 2 *post hoc* changes to the synthesis: *1*) the addition of the “aggregate scores of development” category to capture outcomes that spanned across >1 developmental domain, and *2*) the outcome “social–emotional” was broadened to “social–emotional and behavioral development” to better describe the variety of reported suboutcomes, which aligned more accurately with the terminology used in the 2020 DGAC review. Heterogeneity in population and seafood type were then additionally considered to draw conclusions for each suboutcome within the broader outcome category.

Results are described at the study level because there were cases in which there were multiple articles per study. Two analysts independently reviewed data from each article considering the direction, magnitude, and statistical significance of the reported results, and concluded whether the results (i.e. effects or associations) indicated that there was either *1*) a relationship between higher seafood intake and better neurocognitive development, *2*) a relationship between higher seafood intake and worse neurocognitive development, *3*) no relationship between seafood intake and neurocognitive development, or *4*) reported relationships were inconsistent. Using the study-level conclusions, each analyst then drafted suboutcome-level conclusions across all included studies, and finally, outcome-level conclusions. Discrepancies were resolved through discussion until consensus was reached. For outcomes that evidence support a conclusion, sensitivity analyses were conducted by omitting studies that were at high or very high risk of bias. Data extracted and synthesized for each outcome are described in the Supplemental Data Appendix as well as details of each assessment tool and guidance for interpreting the results are available in column U “Assessment tool interpretation” in each tab. Study characteristics and outcome data are presented in tabular format throughout the manuscript.

### Certainty of evidence

For each conclusion, Grading of Recommendations Assessment, Development and Evaluations (GRADE) was used to assess the certainty of the evidence [18]. GRADE considers risk of bias, inconsistency, indirectness, imprecision, and publication bias. For observational study designs, there can be additional considerations related to dose–response relationships, the magnitude of effect, and residual confounding. RCTs and nonrandomized studies of exposure (i.e. PCSs and retrospective cohort studies) were assessed separately and the overall certainty rating was based on the study design with the highest certainty.

## Results

### Search results

From a total of 1391 records identified in the database search, 40 articles were included that analyzed data from 1 RCT, 24 PCSs, and 1 retrospective cohort study, as described in the next sections (Supplemental Figure 2). This included 14 additional articles [7,[19], [20], [21], [22], [23], [24], [25], [26], [27], [28], [29], [30], [31]] since the previous review [11]. Full-text articles that were reviewed and excluded are listed in Supplemental Table 4.

### Study characteristics

**FIGURE 1:**
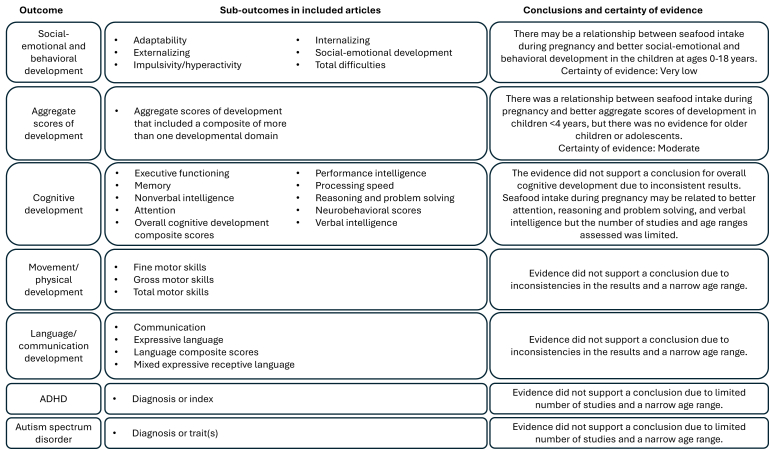
Summary of conclusions for relationships between seafood intake during pregnancy and lactation and neurocognitive development in the children. Certainty of evidence is further described in Table 3 and results of each outcome are described in Tables 2 and 4–9. ADHD, attention-deficit/hyperactivity disorder.

All articles assessed seafood intake during pregnancy; no articles were identified that assessed seafood intake during lactation. The study characteristics of the included articles are in Table 1. In brief, food frequency questionnaires (FFQ) were used most often to assess seafood intake, and assessment timing ranged from 10 wk gestation to a retrospective assessment at 3 mo post delivery. The type of seafood intake varied across studies and included total seafood (inclusive of fish and shellfish), total fish, fatty fish (e.g. oily fish, salmon), specific fish types (e.g. white fish, fried fish, canned tuna, lean fish), and seafood source (e.g. local fish, freshwater, ocean). Age at outcome assessment also varied. Outcomes were measured in children aged 6 mo–17 y via various assessment tools. A summary of conclusions for each outcome is shown in Figure 1 and described next.

**TABLE 1:** Characteristics of studies about relationships between seafood consumption during pregnancy and lactation and child neurocognitive development.

Randomized controlled trials (parallel arm design)					
Study (articles)	Sample characteristics^1^	Seafood intervention	Comparator intervention(s)	Dietary compliance	Funding source
Mommy’s Food Study [22,23]	Pregnant females in Norway and measurements in children aged 3–12 mo; *n* = 133–137	400 g/wk of cod provided as frozen fillets for 16 wk during 20–36 wk gestation	Habitual dietary pattern, seafood intake during intervention not reported	Mothers weighed cod pre- and postmeal to assess grams consumed	The Norwegian Seafood Research Fund

**Nonrandomized cohort studies**					

**Study**	**Sample characteristics**	**Self-reported seafood exposure**		**Dietary assessment method**	**Funding source**

Avon Longitudinal Study of Parents and Children [[32], [33], [34], [35], [36]]	Pregnant females in United Kingdom and measurements in children aged 6 mo–13 y; *n* = 641–8916	Any or higher vs. no or lower servings or amounts of various seafood types including white fish, oily fish, and shellfish at 32 wk gestation		Food frequency questionnaire (FFQ), details and validation not described^2^	UK Medical Research Council; Wellcome Trust; University of Bristol; NOAA; NIAAA; NIH; NIHR; Biomedical Research Centre at the University Hospitals Bristol; NHS Foundation Trust; NICHD; Economic and Social Research Council; Medical Research Council; University of Bristol, UK government departments; Medical Charities; DEE; Nutricia^3^; The Ministry of Agriculture, Foods and Fisheries; Departments of Health and the Environment; South West Regional Health Authority; National Eye Research Centre; Cow and Gate^3^; Milupa,^3^ Scotia Pharmaceuticals,^3^ Stirling^3^
Danish National Birth Cohort (DNBC) [37]	Pregnant females in Denmark and measurements in children aged 6–19 mo; *n* = 92,676	Higher vs. lower g/wk or servings/wk of fish intake at 25 wk gestation		Validated FFQ with standard portion sizes	Danish National Research Foundation; Danish Pharmaceutical Association; Danish Ministry of Health; Danish National Board of Health; Statens Serum Institute; BIOMED; March of Dimes; Danish Heart Association; Danish Medical Research Council; Sygekassernes Helsefond Foundation; Early Nutrition Programming Project, NIH; American Scandinavian Foundation; Inger and Jens Bruun Foundation; Mead Johnson Nutritionals^3^; National Food Producers Association
Early Autism Risk Longitudinal Investigation (EARLI) and/or the Health Outcomes and Measures of the Environment (HOME) Study [28,38]	Pregnant females in the United States and measurements in children aged 3–8 y; *n* = 468–638	Higher vs. lower frequency of seafood including salmon, fatty fish, shellfish, and fried fish during early (20 wk gestation), late (36 wk gestation), and total duration of pregnancy		EARLI: validated FFQ HOME: interview, validation not described	NIEHS; EPA; NIH; NIMH; NICHD; NIND; and Autism Speaks
Infancia y Medio Ambiente [21, 39, 40]	Pregnant females in Spain and measurements in children aged 11 mo–8 y; *n* = 2644–2506	Higher vs. lower amounts of seafood intake including large fatty fish, small fatty fish, lean fish, canned tuna, and shellfish during first trimester (10–13 wk gestation), third trimester (28–32 wk gestation)		FFQ, details and validation not described for all articles	Spanish Institute of Health; Carlos III; Infancia y Medio Ambiente Network grants; Fondo de Investigación Sanitaria; Fondo de Investigación Sanitaria-Fondo Europeo de Desarrollo Regional; Generalitat de Catalunya-Consejo Interdepartamental de Investigación e Innovación Tecnológica; Juan de la Cierva; Conselleria de Sanitat Generalitat Valenciana; Universidad de Oviedo; Obra Social Cajastur; Department of Health of the Basque Government; Provincial Government of Gipuzkoa; Fundación Roger Torné; Ministry of Economy and Competitiveness; Generalitat de Catalunya-CIRIT; Generalitat Valenciana; Alicia Koplowitz Foundation; Fundación Cajastur-Liberbank, Conselleria de Sanitat Generalitat de Catalunya; Diputación Foral de Guipúzcoa; Departamento de Sanidad y Consumo Gobierno Vasco; European Union Sixth Framework Project
Étude Longitudinale Française depuis l’Enfance (ELFE) [19]	Pregnant females in France and measurements in children aged 1–3.5 y; *n* = 18,329	Higher vs. lower frequency of fish intake during the last 3 mo of pregnancy		Validated FFQ	French Institute for Demographic Studies; National Institute of Health and Medical Research; French blood transfusion service; Santé publique France; the National Institute for Statistics and Economic Studies; France, Directorate General of Health France; Ministry for the Environment, France; Ministry of Health and Social Affairs, France; Ministry of Culture, France; National Family Allowance Fund, France; Ministry of Higher Education and Research, France; Institute for Youth and Community Education, France; National Research Agency
Fish Oil and Probiotics in Pregnancy (FOPP) [26]	Pregnant females in Finland and measurements in children aged 2 y; *n* = 439	Higher vs. lower frequency of fish intake during early (14 wk gestation) and late (35 wk gestation) pregnancy		FFQ, details, and validation not described	Academy of Finland; State research funding for university-level health research of the Turku University Hospital; Diabetes Research Foundation; Juho Vainio Foundation; Päivikki and Sakari Sohlberg Foundation; Gyllenberg Foundation; University of Turku
Generation R [41]	Pregnant females in the Netherlands and measurements in children aged 6 y; *n* = 6611	Higher vs. lower fish intake over the prior 3 mo assessed during early pregnancy (median 13.8 wk gestation)		Validated FFQ	Netherlands Organization for Health Research and Development; European Community’s 7th Framework Program; Erasmus Medical Center, Erasmus University; Dutch Ministry of Health, Welfare, and Sport; Netherlands Organization for Health Research and Development
Grass Narrows Community Health Assessment (GN-CHA) [29]	Pregnant females in Canada and measurements in children aged 17 y; *n* = 353	Higher vs. lower frequency of local fish during pregnancy		FFQ, validation not described	Netherlands Organization for Health Research and Development; European Community’s 7th Framework Programme; Erasmus Medical Center, Erasmus University; Dutch Ministry of Health, Welfare, and Sport; Netherlands Organization for Health Research and Development
Japan Environment and Children’s Study [20]	Pregnant females in Japan and measurements in children aged 6 mo–1 y; *n* = 104,065	Higher vs. lower amounts of fish intake during mid/late pregnancy		Validated FFQ	First Bank of Toyama Scholarship Foundation; the DHA&EPA Association; Niigata Medical Association; Toyama Medical Association, Toyama; Occupational Health Promotion Center; Otsuka Pharmaceuticals^3^
Laizhou Wan Birth Cohort [42]	Pregnant females in China and measurements in children aged 1 y; *n* = 566	Higher vs. lower frequency of total fish intake during pregnancy assessed after delivery		FFQ, validation not described	Natural Science Foundation of China; National Basic Research Program of China; Science and Technology Commission of Shanghai Municipality;
Public Health Impact of long-term, low level, mixed element exposure in susceptible population strata (PHIME) [24, 43]	Pregnant females in Italy, Slovenia, Croatia, and Greece and measurements in children aged 18 mo; *n* = 2189	Higher vs. lower amounts of fish intake during pregnancy including fish, crustaceans, and mollusks, and fish in oil assessed after delivery		Validated FFQ but adapted from original	European Union; Slovenian Agency for Research; Institute for Maternal and Child Health; Italian Ministry of Health; European Commission; University of Rijeka
Mount Sinai Children’s Environmental Health Study [44]	Pregnant females in the United States and measurements in children aged 18 mo–9 y; *n* = 404	Higher vs. lower frequency of canned fish intake during third trimester of pregnancy		Single question, validation not described	NIEHS; EPA; New York Community Trust, Agency for Toxic Substances and Disease Registry; CDC; Association of Teachers of Preventive Medicine
Norwegian Mother and Child Cohort Study (MoBa) [45]	Pregnant females in Norway and measurements in children aged 5 y; *n* ∼ 39,000	Higher vs. lower amounts of seafood including fish, shellfish, and crustaceans assessed at 22 wk gestation		Validated FFQ	Norwegian Ministry of Health and Care Services; Ministry of Education and Research; NIH; NIEHS; NINDS
Odense Child Cohort [31]	Pregnant females in Denmark and measurements in children aged 20–36 mo; *n* = 2448	Higher vs. lower frequency of fish intake (not described)		Single question, validation not described	Novo Nordic Foundation; Danish Council for Independent Research Odense University Hospital
Project Viva [[46], [47], [48]]	Pregnant females in the United States and measurements in children aged 6 mo–8 y; *n* = 896–2128	Higher vs. lower frequency of fish including canned tuna fish, shrimp, lobster, scallops, clams, dark-meat fish (e.g. mackerel, salmon, sardines, bluefish, swordfish), and other fish (e.g. cod, haddock, halibut) during second trimester		Validated FFQ modified for pregnant females and calibrated against erythrocyte levels of elongated *n* – 3 fatty acids	NIAAA; Harvard Medical School; Harvard Pilgrim Health Care Foundation
Nutrition Cohort 1 of the Seychelles Child Development Study^4^ [7, 49]	Pregnant females in the Seychelles and measurements in children aged 9–30 mo ≤9 y; *n* = 300	Higher vs. lower amounts of fatty and lean fish at 28 wk gestation		FFQ and 4-d food diary on 2 consecutive weekdays and 2 weekend days, validation not described	NIEHS; Government of Seychelles
The New Bedford Cohort [50]	Pregnant females in the United States and measurements in children aged 8 y; *n* = 788	Higher vs. lower frequency of fish intake including dark fish (e.g. salmon, mackerel, bluefish, and swordfish), tuna (including canned tuna), shellfish (e.g. lobster and clams), eel, and other fish assessed after delivery		FFQ, validation not described	NIEHS
Unnamed cohort in China [25]	Pregnant females in China and measurements in children aged 12 and 36 mo; *n =* 408	Higher vs. lower amounts of fish intake including freshwater fish, ocean fish, shrimp, eel, snails, crab, and other shellfish during the third trimester of pregnancy (between 4 wk before delivery or 1 wk postpartum)		Validated FFQ	NIEHS; U.S. National Institute of Health Loan Replacement Program; National Natural Science Foundation of China
Unnamed cohort in Finland [51]	Pregnant females in Finland and measurements in children aged 2 y; *n =* 256	Higher vs. lower frequency of fish over past 2 wk during third trimester		FFQ, validation not described	Academy of Finland; Social Insurance Institution of Finland
Unnamed cohort(s)^5^ in Italy [30, 52,53]	Pregnant females in Northeast Italy and measurements in children aged 18 mo–7 y; *n =* 242–900	Higher vs. lower frequency of seafood intake including fish (fresh fish and carnivorous fish such as eel, gilthead bream, sea bass, angler fish, John dory), crustaceans, mollusks, tuna, mackerel, and sardines in oil assessed after delivery		Validated FFQs but adapted from original use or structured interviews	European Union; Slovenian Research Agency; Institute for Maternal and Child Health; Italian Ministry of Health; Region Friuli Venezia Giulia
Unnamed cohort in Japan [54]	Pregnant females in Japan and measurements in children aged 18 mo; *n =* 315	Higher vs. lower frequency of fish (not described) intake during pregnancy		FFQ, details, and validation not described	Not reported
Unnamed cohort in Norway [27]	Pregnant females in Norway and measurements in children aged 6 mo; *n =* 140	Higher vs. lower frequency of fish intake for dinner at 18-, 28-, and 36-wk’ gestation		FFQ, details, and validation not described	Department of Laboratory Medicine, University Hospital of North Norway; North Norway Regional Health Authority; Department of Medical Biochemistry and Pharmacology at Haukeland University Hospital
Unnamed cohort in Spain [55]	Pregnant females in Spain and measurements in children aged 4 y; *n =* 482	Higher vs. lower frequency of seafood including fish, squid, and shellfish assessed 3 mo after delivery		Semiquantitative FFQ administer via interview, validation not described	Spanish Ministry of Health; Instituto de Salud Carlos III; “Fundació La Caixa”; European Commission; European Union
Unnamed cohort in the UK [56]	Pregnant females in the UK and measurements in children aged 9 y; *n =* 217	Higher vs. lower frequency over past 3 mo of white fish (grilled, poached, steamed, in crumbs or batter), fish pie, fish fingers, fish in sauces, oily fish (e.g. tuna, sardines, trout, salmon, mackerel) and shellfish (e.g. crab, prawns, mussels) during 15- and 32-wk’ gestation		FFQ, validation not described	Medical Research Council; WellChild
Unnamed cohort in the United States [57]	Pregnant females in the UK and measurements in children aged 9 y; *n =* 329	Fish vs. no fish intake during pregnancy assessed post-partum		Interview, validation not described	September 11th Fund of the New York Community Trust; United Way of New York City; New York Times 9/11 Neediest Fund; National Philanthropic Trust; NIEHS; EPA

Abbreviations: CDC, Centers for Disease Control and Prevention; CIRIT, Consell Interdepartamental de Recerca i Innovació Tecnológica; DEE, Department of Education and Employment; FFQ, food frequency questionnaire; NHS, National Health Service; NIAAA, National Institute on Alcohol Abuse and Alcoholism; NICHD, National Institute of Child Health and Human Development; NIEHS, National Institute of Environmental Health Sciences; NIHR, National Institute for Health and Care Research United Kingdom; NIMH, National Institute of Mental Health; NINDS, National Institute of Neurologic Disease; NOAA, National Oceanic and Atmospheric Administration; United States EPA, United States Environmental Protection Agency.

1 Sample size and age of children may vary for different outcomes reported in the article.

2 Validation as reported by authors. This does not mean that the assessment tool was validated to assess seafood intake specifically.

3 Name of a for-profit entity.

4 Presumably both articles are from the Nutrition Cohort 1 based on recruitment date, location, and study and participant characteristics.

5 It was unclear if these articles were written about the same cohort. The location and timing of data collection were similar, so these were considered as potentially from the same cohort to be conservative over concerns of multiplicity.

### Social–emotional and behavioral development

**FIGURE 2:**
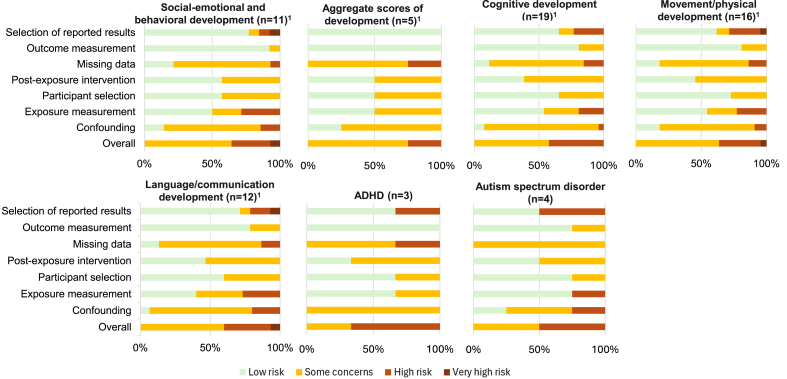
Risk of bias of included articles. *n =* the total number of studies included in that outcome category, including prospective cohorts, retrospective cohorts, and randomized controlled trials. ^1^Risk of bias was assessed with ROBINS-E, except one of studies included in this count was a randomized controlled trial (RCT) that was assessed using ROB 2.0. Those results are presented in Supplemental Tables 5–11 for relevant outcomes. In brief, this RCT had Some Concerns of bias overall because of deviation from intended intervention assessed with ROB 2.0. RCT, randomized controlled trial; ROB 2.0, version 2 of the Cochrane risk-of-bias tool for randomized trials. ADHD, attention-deficit/hyperactivity disorder.

There was 1 RCT [22], 1 retrospective cohort [29], and 9 PCSs [7,20,30,[32], [33], [34],^37^,^42^,^44^,^50^,^52^,^56^] conducted in 9 different countries that assessed relationships between seafood intake during pregnancy and social–emotional and behavioral development outcomes in the children aged 3 mo–17 y. Nine articles had some concerns of bias, 4 were at high risk due to confounding, exposure measurement, and missing data, and 1 study was at very high risk due to reporting bias (Figure 2 and Supplemental Table 5).

Results were consistent across study designs for social–emotional and behavioral development outcomes (Table 2). The 1 RCT showed lower social–emotional problems at 3–6 mo when mothers were provided 400g of cod/wk compared with habitual dietary patterns for 16 wk during 20–36 wk gestation [22]. These results were supported by cohort studies that largely suggested higher seafood intake during pregnancy was related to better social–emotional and behavioral development outcomes in the children aged 3 mo–17 y. For social–emotional and behavioral development suboutcomes, the evidence suggested that there may be relationships between higher seafood intake and better adaptability (3 of 3 studies), externalizing (2 of 2 studies), impulsivity/hyperactivity (3 of 4 studies), and social–emotional development (4 of 7 studies) outcomes. At least half of the studies within each suboutcome reported ≥1 statistically significant result. However, results were inconsistent for internalizing behaviors and total behavioral difficulties. There were 2 studies that suggested higher seafood was related to worse social–emotional and behavioral development outcomes, but 1 was a retrospective (rather than prospective) cohort study [29] and the other assessed canned tuna [44] which is high in mercury, potentially contributing to the opposite direction of results. Both articles were also at high risk for bias. Overall, the evidence suggested that there may be a relationship between seafood intake of ∼4–16 oz/wk during pregnancy and better social–emotional and behavioral development outcomes in the children aged 0–18 y. The certainty of evidence was very low (Table 3).

**TABLE 2:** Seafood during pregnancy and social–emotional and behavioral development outcomes in the children.

Suboutcome	Study name (references)^1^	Overall risk of bias	Age at outcome assessment	Number of assessments	Study conclusion	Suboutcome conclusion	Outcome conclusion
Adaptability	Laizhou Wan Birth Cohort [42]	**Some concerns**	1 y	1	Favors higher seafood∗	Favors higher seafood	Favors higher seafood
	Unnamed cohort in Italy [52]	**High**	18 mo	1	Favors higher seafood∗		

Mount Sinai Children’s Environmental Health Study [44]	**High**	4–9 y	1	Favors higher seafood			

Externalizing	Avon Longitudinal Study of Parents and Children [33]	**Some concerns**	4–13 y	10	Favors higher seafood∗	Favors higher seafood	

Unnamed cohort in the United Kingdom (UK) [56]	**Some concerns**	9 y	2	Favors higher seafood			

Impulsivity/hyperactivity	The New Bedford Cohort [50]	**High**	8 y	1	Favors higher seafood∗	Favors higher seafood	

	Unnamed cohort in the UK [56]	**Some concerns**	9 y	2	Favors higher seafood∗		

Avon Longitudinal Study of Parents and Children [33]	**Some concerns**	7 y	1	Favors higher seafood			

Mount Sinai Children’s Environmental Health Study [44]	**High**	4–9 y	1	Neither			

Internalizing	Avon Longitudinal Study of Parents and Children [33,34]	**Some concerns**	4–13 y	5	Favors higher seafood∗	No conclusion	

	Unnamed cohort in the UK [56]	**Some concerns**	9 y	4	Inconsistent		

Mount Sinai Children’s Environmental Health Study [44]	**High**	4–9 y	1	Favors lower seafood			

Social–emotional development	Avon Longitudinal Study of Parents and Children [33,34]	**Some concerns**	6 mo to 7 y	11	Favors higher seafood∗	Favors higher seafood

	Danish National Birth Cohort [37]	**Some concerns**	6 mo	1	Favors higher seafood∗	

	Mommy's Food Study^2^ [22]	**Some concerns**	3–6 mo	2	Favors higher seafood∗	

	Japan Environment and Children’s Study [20]	**Some concerns**	6 mo–1 y	2	Favors higher seafood	

Laizhou Wan Birth Cohort [42]	**Some concerns**	1 y	1	Neither

Grass Narrows Community Health Assessment (GN-CHA)^3^ [29]	**High**	0–17 y	2	Favors lower seafood∗

Unnamed cohort(s) in Italy [30,52]	**High/Very High**	18 mo	4	Unclear

Total difficulties	Avon Longitudinal Study of Parents and Children [33]	**Some concerns**	7 y	1	Favors higher seafood	No conclusion
	Nutrition Cohort 1 of the Seychelles Child Development Study^4^ [7]	**Some concerns**	5–9 y	4	Inconsistent	

Unnamed cohort in the UK [56]	**Some concerns**	9 y	3	Inconsistent

Two analysts independently reviewed data from each article considering the direction, magnitude, and statistical significance of the reported results, and concluded whether the results indicated that there was either *1*) a relationship between higher seafood intake and better neurocognitive development (“favors higher seafood”), *2*) a relationship between higher seafood intake and worse neurocognitive development (“favors lower seafood”), *3*) no relationship between seafood intake and neurocognitive development (“neither”), or *4*) reported relationships were inconsistent (“inconsistent”). “Unclear” indicates that the data were not reported to determine direction and magnitude of effects or associations. Using the study-level conclusions, each analyst then drafted a suboutcome-level conclusion, and finally, outcome-level conclusions. Discrepancies were resolved through discussion.

∗ Indicates that ≥1 of the results within that suboutcome for that study was statistically significant.

1 All studies are prospective cohort studies unless otherwise indicated.

2 Randomized controlled trial.

3 Retrospective cohort study.

4 Presumably is Nutrition Cohort 1 based on recruitment date, location, and study and participant characteristics.

**TABLE 3:** Certainty of evidence ratings using GRADE^1^ [18] by outcome and by study design.

Social–emotional and behavioral development: The evidence suggested that there may be a relationship between seafood intake during pregnancy and better social–emotional and behavioral development outcomes in the children aged 0–18 y (overall certainty rating^1^: very low).									
Study design; no. of articles	Risk of bias^2^	Inconsistency^3^	Indirectness	Imprecision^4^	Publication bias^5^	Large effect	Plausible confounding	Dose–response	Certainty
Summary of findings for randomized controlled trials (RCTs): Higher vs. lower seafood intake for 16 wk during pregnancy resulted in lower social–emotional and behavioral development problems in the children aged 3–6 mo.									
*n =* 1 RCT [22]	Not serious	Not applicable; only 1 study	Very serious; 0–2 y in Norway; only cod assessed; behavior limited to social–emotional problems	Not serious	Strongly detected; only 1 article	Not applicable	Not applicable	Not applicable	Very low
Summary of findings for prospective cohort studies (PCSs): The evidence suggested a relationship between seafood intake during pregnancy and better social–emotional and behavioral development outcomes in the children aged 0–17 y.									
*n =* 13 cohort studies [7,20,29,30,[44], [45], [46], [47], [48], [49], [50], [51], [52], [53], [54], [55], [56]]	Very serious; most high ROB, 1 very high	Not serious	Not serious	Not serious	Undetected	No	No	No	Low

**Aggregate scores of development:The evidence did not support a conclusion about seafood intake during pregnancy and aggregate scores of development in the children aged 0–18 y. However, there was a relationship between seafood intake during pregnancy and better aggregate scores of development in children <4 y old (overall certainty rating: moderate).**									

**Study design; no. of articles**	**Risk of bias**^2^	**Inconsistency**^3^	**Indirectness**	**Imprecision**^4^	**Publication bias**^**5**^	**Large effect**	**Plausible confounding**	**Dose–response**	**Certainty**

Summary of findings from RCTs: Higher vs. lower seafood consumption for 16 wk during pregnancy resulted in no differential effects on aggregate scores of development in the children aged 3–12 mo.									
*n =* 1 RCT [22]	Not serious	Not applicable; only 1 study	Not serious	Not serious	Strongly detected; only 1 article	Not applicable	Not applicable	Not applicable	Moderate
Summary of findings from PCSs: The evidence suggested a relationship between seafood intake during pregnancy and better aggregate scores of development in the children aged <4 y.									
*n =* 4 [19,34,37,54]	Not serious; all some concerns, only 1 at high risk	Not serious	Not serious; all <4 y	Not serious, 4 of 5 studies reported ≥1 statistically significant result	Strongly detected; 4 of 5 studies in same direction, all with ≥1 statistically significant result	No	No	No	Moderate

Abbreviations: GRADE, Grading of Recommendations, Assessment, Development, and Evaluation; ROB, risk of bias; RCT, randomized controlled trial; NRS-Exp, non-randomized study of exposure; n/a, not applicable.

1 GRADE rating: very low, low, moderate, or high. All studies were considered nonrandomized study of exposures according to the GRADE framework.

2 Domain only downgrades. Rating choices: extremely serious, very serious, serious, or not serious.

3 Domain only downgrades. Rating choices: very serious, serious, or not serious.

4 Domain only downgrades. Rating choices: strongly detected, or undetected.

Omitting articles that were at high or very high risk of bias did not change the conclusion because this omitted the studies either suggested that higher seafood intake was associated with worse social–emotional and behavioral development outcomes [29,44] or the direction or magnitude of associations were not reported [30,52].

### Aggregate scores of developments

There was 1 RCT [22] and 4 PCSs [19,34,37,54] conducted in 5 countries that assessed relationships between seafood intake during pregnancy and aggregate scores of development in children aged 3 mo–3.5 y (Table 4). Four articles had some concerns for bias, and 1 was at high risk of bias due to missing data (Supplemental Table 6).

**TABLE 4:** Seafood during pregnancy and aggregate scores of development outcomes in the children.

Study name (references)^1^	Risk of bias	Age at outcome assessment	Number of assessments	Study conclusion	Outcome conclusion
Avon Longitudinal Study of Parents and Children [34]	**Some concerns**	18 mo	3	Favors higher seafood∗	Favors higher seafood
Danish National Birth Cohort [37]	**Some concerns**	6–19 mo	5	Favors higher seafood∗	

Étude Longitudinale Française depuis l’Enfance (ELFE) [19]	**Some concerns**	1–3.5 y	3	Favors higher seafood∗	

Unnamed cohort in Japan [54]	**High**	18 mo	5	Favors higher seafood∗

Mommy's Food Study^2^ [22]	**Some concerns**	3–12 mo	3	Neither

Two analysts independently reviewed data from each article considering the direction, magnitude, and statistical significance of the reported results, and concluded whether the results indicated that there was either *1*) a relationship between higher seafood intake and better neurocognitive development (“favors higher seafood”), *2*) a relationship between higher seafood intake and worse neurocognitive development (“favors lower seafood”), *3*) no relationship between seafood intake and neurocognitive development (“neither”), or *4*) reported relationships were inconsistent (“inconsistent”). "Unclear" indicates that the data were not reported to determine direction and magnitude of effects or associations. Using the study-level conclusions, each analyst then drafted a suboutcome-level conclusion, and finally, outcome-level conclusions. Discrepancies were resolved through discussion.

∗ Indicates that ≥1 of the results within that suboutcome for that study was statistically significant.

1 All studies are prospective cohort studies unless otherwise indicated.

2 Randomized controlled trial.

The 1 RCT showed no differential effect on aggregate scores of development at 3–12 mo when mothers were provided 400 g of cod/wk compared with habitual dietary patterns during pregnancy. However, all 4 PCSs suggested a relationship between higher seafood intake and better aggregate scores of development for children aged <4 y, all of which reported ≥1 statistically significant result. There was no evidence identified for older children or adolescents. Overall, the evidence suggested a relationship between higher seafood intake and better aggregate scores of development for children aged <4 y (certainty of evidence: moderate; Table 3), but there was no evidence to support a conclusion that could be generalized to ages 0–18.

Omitting an article from 1 PCS that was at high risk of bias did not change the conclusions because the results were consistent in magnitude, direction, and statistical significance across all PCSs.

### Cognitive development

There was 1 RCT [23] and 18 PCSs [7,[19], [20], [21],[24], [25], [26],^28^,^33^,[38], [39], [40], [41],^43^,^44^,[46], [47], [48], [49], [50],^52^,^53^,[55], [56], [57]] conducted in 14 countries that assessed relationships between seafood intake during pregnancy and cognitive development in the children aged 5 wk–9 y. Fifteen articles had some concerns for bias and 11 were at high risk of bias due to confounding, exposure measurement, missing data, and selective reporting of findings (Figure 2 and Supplemental Table 7).

Results were inconsistent across the 10 cognitive development suboutcomes, in which 3 suggested a potentially beneficial relationship and 7 were unclear (Table 5). The evidence suggested a relationship between higher seafood intake and better attention (3 of 3 studies), reasoning and problem-solving (4 of 6 studies), and verbal intelligence (5 of 9 studies) outcomes. Most studies reported ≥1 statistically significant result in favor of seafood within those suboutcomes. However, results were inconsistent for executive functioning, memory, nonverbal intelligence, overall cognitive development composite scores, processing speed, performance intelligence, and neurobehavioral outcomes. Additionally, there were 4 studies that suggested a potential relationship between higher seafood intake and worse cognitive development outcomes. Two of these 4 assessed canned tuna [44,53] which is high in mercury, and 1 reported that shellfish and squid (but not fish) was associated with lower cognitive scores [55]. Overall, the inconsistent evidence and narrow age range did not support a conclusion between seafood intake during pregnancy and overall cognitive development in the child. Seafood intake during pregnancy may be beneficial for certain cognitive development suboutcomes in children, but the evidence was too limited in the number of studies and age groups assessed to draw conclusions.

**TABLE 5:** Seafood during pregnancy and cognitive development outcomes in the children.

Suboutcome	Study name (references)^1^	Overall risk of bias	Age at outcome assessment	Number of assessments	Study conclusion	Suboutcome conclusion	Outcome conclusion
Executive functioning	Infancia y Medio Ambiente [39]	**High**	5 y	1	Favors higher seafood∗	No conclusion	No conclusion
	Mount Sinai Children’s Environmental Health Study [44]	**High**	4–9 y	1	Favors lower seafood		

Memory	Infancia y Medio Ambiente [39]	**High**	5 y	1	Favors higher seafood∗	No conclusion	

	Nutrition Cohort 1 of the Seychelles Child Development Study^2^ [7,49]	**Some concerns**	25 mo–9 y	5	Favors higher seafood		

Unnamed cohort in Spain [55]	**High**	4 y	3	Inconsistent			

Project Viva [46,47]	**Some concerns**	6 mo–8 y	10	Inconsistent			

Nonverbal intelligence	Infancia y Medio Ambiente [39]	**High**	5 y	1	Favors higher seafood∗	No conclusion	

	Generation R [41]	**Some concerns**	6 y	2	Inconsistent		

Project Viva [46]	**Some concerns**	8 y	3	Inconsistent			

Unnamed cohort in Spain [55]	**High**	4 y	3	Favors lower seafood∗			

Attention	Infancia y Medio Ambiente [21]	**High**	8 y old	12	Favors higher seafood∗	Favors higher seafood	

	Nutrition Cohort 1 of the Seychelles Child Development Study^2^ [7,49]	**Some concerns**	9 mo–9 y	13	Favors higher seafood		

The New Bedford Cohort [50]	**High**	8 y	6	Favors higher seafood			

Overall cognitive development composite scores	Avon Longitudinal Study of Parents and Children [33]	**Some concerns**	8 y	1	Favors higher seafood∗	No conclusion	

	Infancia y Medio Ambiente [39,40]	**High/ Some concerns**	14 mo–5 y	34	Favors higher seafood∗		

	Unnamed cohort in China [25]	**Some concerns**	12–36 mo	1	Favors higher seafood∗	

	Unnamed cohort in the United States [57]	**High**	12–48 mo	4	Favors higher seafood∗	

	Unnamed cohort(s) in Italy [52, 53]	**High**	18 mo–7 y	3	Favors higher seafood	

	Early Autism Risk Longitudinal Investigation (EARLI) and the Health Outcomes and Measures of the Environment (HOME) Study [28]	**Some concerns**	3 y	29	Inconsistent	

Nutrition Cohort 1 of the Seychelles Child Development Study^2^ [7,49]	**Some concerns**	9 mo–9 y	10	Inconsistent

Public Health Impact of long-term, low level, Mixed Element exposure in susceptible population strata (PHIME) [24, 43]	**High**	18 mo	3	Inconsistent

Unnamed cohort in the United Kingdom [56]	**Some concerns**	9 y	4	Inconsistent

Mommy's Food Study^3^ [23]	**Some concerns**	11 mo	1	Favors lower seafood∗

Unnamed cohort in Spain [55]	**High**	4 y	4	Favors lower seafood∗

Fish Oil and Probiotics in Pregnancy (FOPP) [26]	**High**	2 y	2	Unclear

Performance intelligence	Unnamed cohort in Italy [53]	**High**	2–9 y	2	Favors higher seafood	No conclusion

	Unnamed cohort in the United States [57]	**High**	2 y	1	Favors higher seafood	

Avon Longitudinal Study of Parents and Children [33]	**Some concerns**	8 y	1	Neither

Unnamed cohort in the UK [56]	**Some concerns**	9 y	4	Unclear

Processing speed	The New Bedford Cohort [50]	**High**	8 y	1	Favors higher seafood	No conclusion	
	Nutrition Cohort 1 of the Seychelles Child Development Study^2^ [7]	**Some concerns**	9 y	2	Inconsistent		

Mount Sinai Children’s Environmental Health Study [44]	**High**	4–9 y	1	Favors lower seafood			

Reasoning and problem-solving	Infancia y Medio Ambiente [39]	**High**	5 y	1	Favors higher seafood∗	Favors higher seafood	

	Japan Environment and Children’s Study [20]	**Some concerns**	6 mo–1 y	2	Favors higher seafood∗		

	Mount Sinai Children’s Environmental Health Study [33]	**High**	4–9 y	1	Favors higher seafood∗		

Étude Longitudinale Française depuis l’Enfance (ELFE) [19]	**Some concerns**	3.5 y	1	Favors higher seafood			

Nutrition Cohort 1 of the Seychelles Child Development Study^2^ [7]	**Some concerns**	5 y	4	Inconsistent			

Unnamed cohort in Spain [55]	**High**	4 y	3	Inconsistent			

Neurobehavioral	Health Outcomes and Measures of the Environment (HOME) Study [38]	**High**	5 wk	4	Inconsistent	No conclusion	

Fish Oil and Probiotics in Pregnancy (FOPP) [26]	**Some concerns**	2 y	4	Unclear			

Verbal intelligence	Avon Longitudinal Study of Parents and Children [33]	**Some concerns**	8 y	1	Favors higher seafood∗	Favors higher seafood
	Infancia y Medio Ambiente [39]	**High**	5 y	1	Favors higher seafood∗	

	Project Viva [46, 48]	**Some concerns**	3–8 y	7	Favors higher seafood∗	

	Unnamed cohort in the UK [56]	**Some concerns**	9 y	5	Favors higher seafood∗	

	Unnamed cohort in the United States [57]	**High**	2 y	1	Favors higher seafood∗	

Nutrition Cohort 1 of the Seychelles Child Development Study^2^ [7]	**Some concerns**	5–9 y	4	Inconsistent

Unnamed cohort in Spain [55]	**High**	4 y	3	Inconsistent

Mount Sinai Children’s Environmental Health Study [44]	**High**	4–9 y	1	Favors lower seafood

Unnamed cohort in Italy [53]	**High**	7 y	2	Favors lower seafood

Two analysts independently reviewed data from each article considering the direction, magnitude, and statistical significance of the reported results, and concluded whether the results indicated that there was either *1*) a relationship between higher seafood intake and better neurocognitive development (“favors higher seafood”), *2*) a relationship between higher seafood intake and worse neurocognitive development (“favors lower seafood”), *3*) no relationship between seafood intake and neurocognitive development (“neither”), or *4*) reported relationships were inconsistent (“inconsistent”). “Unclear” indicates that the data were not reported to determine direction and magnitude of effects or associations. Using the study-level conclusions, each analyst then drafted a suboutcome-level conclusion, and finally, outcome-level conclusions. Discrepancies were resolved through discussion.

∗ Indicates that ≥1 of the results within that suboutcome for that study was statistically significant.

1 All studies are prospective cohort studies unless otherwise indicated.

2 Presumably is Nutrition Cohort 1 based on recruitment date, location, and study and participant characteristics.

3 Randomized controlled trial.

### Movement/physical development

There was 1 RCT [23] and 15 PCSs [7,20,[24], [25], [26], [27],^30^,^33^,^35^,^37^,^39^,^40^,^42^,^43^,^46^,^48^,^49^,^51^,^52^,^55^,^57^] in 13 different countries that assessed relationships between seafood intake during pregnancy and movement/physical development in the children aged 6 mo–9 y. Fourteen articles had some concerns of bias, 7 were at high risk due to confounding, exposure measurement, missing data, or reporting bias and 1 study was at very high risk due to reporting bias (Figure 2 and Supplemental Table 8).

Results were inconsistent within all 3 suboutcomes assessed (Table 6). Within fine motor, gross motor, and total motor skills, less than half of the studies suggested a relationship between higher seafood intake and better motor skills. The rest of the studies were either inconsistent or did not suggest a directional relationship, and 1–2 studies within each suboutcome suggested a relationship between higher seafood and worse motor skills. The inconsistent evidence and narrow age range did not support a conclusion on seafood intake during pregnancy and movement/physical development in the children.

**TABLE 6:** Seafood during pregnancy and movement/physical development outcomes in the children.

Suboutcome	Study name (references)^1^	Overall risk of bias	Age at outcome assessment	Number of assessments	Study conclusion	Suboutcome conclusion	Outcome conclusion
Fine motor	Avon Longitudinal Study of Parents and Children [33]	**Some concerns**	6–42 mo	4	Favors higher seafood∗	No conclusion	No conclusion
	Japan Environment and Children’s Study [20]	**Some concerns**	6 mo–1 y	2	Favors higher seafood∗		

	Laizhou Wan Birth Cohort [42]	**Some concerns**	6 mo–1 y	1	Neither		

	Mommy's Food Study^2^ [23]	**Some concerns**	11 mo	1	Neither		

	Project Viva [46, 48]	**Some concerns**	3–8 y	12	Inconsistent		

Public Health Impact of long-term, low-level, mixed element exposure in susceptible population strata (PHIME) [24, 53]	**High**	18 mo	3	Inconsistent			

Unnamed cohort in Italy [30]	**Very High**	25 mo	5	Favors lower seafood			

Fish Oil and Probiotics in Pregnancy (FOPP) [26]	**Some concerns**	2 y	2	Unclear			

Gross motor	Project Viva [48]	**Some concerns**	3 y	5	Favors higher seafood∗	No conclusion	

	Unnamed cohort in Finland [51]	**Some concerns**	2 y	3	Favors higher seafood		

	Laizhou Wan Birth Cohort [42]	**Some concerns**	1 y	1	Neither		

	Mommy's Food Study^2^ [23]	**Some concerns**	11 mo	1	Neither		

	Avon Longitudinal Study of Parents and Children [33, 35]	**Some concerns**	6 mo–3.5 y	7	Inconsistent		

	Japan Environment and Children’s Study [20]	**Some concerns**	6 mo–1 y	2	Inconsistent		

Public Health Impact of long-term, low-level, Mixed Element exposure in susceptible population strata (PHIME) [24, 43]	**High**	18 mo	3	Inconsistent			

Nutrition Cohort 1 of the Seychelles Child Development Study^3^ [7]	**Some concerns**	9 y	2	Favors lower seafood

Unnamed cohort in Norway [27]	**High**	6 mo	2	Favors lower seafood∗

Fish Oil and Probiotics in Pregnancy (FOPP) [26]	**Some concerns**	2 y	2	Unclear

Unnamed cohort in Italy [30]	**Very High**	25 mo	3	Unclear

Total motor	Infancia y Medio Ambiente [39,40]	**High/Some Concerns**	14 mo–5 y	13	Favors higher seafood∗	No conclusion
	Unnamed cohort in China [25]	**Some concerns**	12–36 mo	1	Favors higher seafood∗	

	Unnamed cohort in the United States [57]	**High**	12–36 mo	3	Favors higher seafood∗	

	Danish National Birth Cohort [37]	**Some concerns**	6–19 mo	2	Favors higher seafood	

	Nutrition Cohort 1 of the Seychelles Child Development Study^3^ [7,49]	**Some concerns**	9–30 mo	6	Inconsistent	

	Public Health Impact of long-term, low level, mixed element exposure in susceptible population strata (PHIME) [24, 43]	**High**	18 mo	3	Inconsistent	
Mommy's Food Study^2^ [23]	**Some concerns**	11 mo	1	Favors lower seafood

Unnamed cohort in Spain [55]	**High**	4 y	3	Favors lower seafood∗

Fish Oil and Probiotics in Pregnancy (FOPP) [26]	**Some concerns**	2 y	2	Unclear

Unnamed cohort in Italy [52]	**High**	18 mo	1	Unclear

Two analysts independently reviewed data from each article considering the direction, magnitude, and statistical significance of the reported results, and concluded whether the results indicated that there was either *1*) a relationship between higher seafood intake and better neurocognitive development (“favors higher seafood”), *2*) a relationship between higher seafood intake and worse neurocognitive development (“favors lower seafood”), *3*) no relationship between seafood intake and neurocognitive development (“neither”), or *4*) reported relationships were inconsistent (“inconsistent”). “Unclear” indicates that the data were not reported to determine direction and magnitude of effects or associations. Using the study-level conclusions, each analyst then drafted a suboutcome-level conclusion, and finally, outcome-level conclusions. Discrepancies were resolved through discussion.

∗ Indicates that ≥1 of the results within that suboutcome for that study was statistically significant.

1 All studies are prospective cohort studies unless otherwise indicated.

2 Randomized controlled trial.

3 Presumably is Nutrition Cohort 1 based on recruitment date, location, and study and participant characteristics.

### Language/communication development

There was 1 RCT [23] and 11 PCS [7,19,20,24,26,30,31,33,34,42,43,45,48,52] conducted in 13 countries that assessed relationships between seafood intake during pregnancy and language/communication development in the children aged 6 mo–9 y. Nine articles had some concerns of bias, 5 were at high risk due to confounding, missing data, exposure measurement, and reporting bias and 1 study was at very high risk of bias due to selective reporting of findings (Figure 2 and Supplemental Table 9).

Results were inconsistent across language/communication development suboutcomes (Table 7). The evidence suggested that there may be a relationship between higher seafood intake during pregnancy and better communication (2 of 3 studies) and expressive language (5 of 9 studies) outcomes in the child, but results were inconsistent for language composite and mixed expressive receptive language. The inconsistent evidence and narrow age range assessed did not support a conclusion between seafood intake during pregnancy and language/communication development outcomes in the children.

**TABLE 7:** Seafood during pregnancy and language/communication development outcomes in the children.

Suboutcome	Study name (references)^1^	Overall risk of bias	Age at outcome assessment	Number of assessments	Study conclusion	Suboutcome conclusion	Outcome conclusion
Communication	Avon Longitudinal Study of Parents and Children [33]	**Some concerns**	6–18 mo	2	Favors higher seafood∗	Favors higher seafood	No conclusion
	Norwegian Mother and Child Cohort Study (MoBa) [45]	**High**	5 y	3	Favors higher seafood∗		

Japan Environment and Children’s Study [20]	**Some concerns**	6 mo–1 y	2	Inconsistent			

Expressive language	Avon Longitudinal Study of Parents and Children [34]	**Some concerns**	15 mo	3	Favors higher seafood∗	Favors higher seafood	

	Fish Oil and Probiotics in Pregnancy (FOPP) [26]	**Some concerns**	2 y	2	Favors higher seafood∗		

	Norwegian Mother and Child Cohort Study (MoBa) [45]	**High**	5 y	3	Favors higher seafood∗		

	Étude Longitudinale Française depuis l’Enfance (ELFE) [19]	**Some concerns**	2 y	1	Favors higher seafood		

	Nutrition Cohort 1 of the Seychelles Child Development Study^2^ [7]	**Some concerns**	5–9 y	6	Favors higher seafood		

Mommy's Food Study^3^ [23]	**Some concerns**	11 mo	1	Neither			

Project Viva [48]	**Some concerns**	3 y	4	Inconsistent			

Public Health Impact of long-term, low level, mixed element exposure in susceptible population strata (PHIME) [24, 43]	**High**	18 mo	3	Inconsistent			

Odense Child Cohort [31]	**High**	21 mo	1	Favors lower seafood			

Language composite scores	Avon Longitudinal Study of Parents and Children [34]	**Some concerns**	18 mo	3	Favors higher seafood∗	No conclusion	

	Norwegian Mother and Child Cohort Study (MoBa) [45]	**High**	5 y	2	Favors higher seafood∗		

	Odense Child Cohort [31]	**High**	30 mo	1	Favors higher seafood∗	

	Public Health Impact of long-term, low-level, mixed element exposure in susceptible population strata (PHIME) [24]	**High**	18 mo	1	Favors higher seafood∗	

	Laizhou Wan Birth Cohort [42]	**Some concerns**	1 y	1	Neither	

Mommy's Food Study^3^ [23]	**Some concerns**	11 mo	1	Neither

Nutrition Cohort 1 of the Seychelles Child Development Study^2^ [7]	**Some concerns**	5 y	2	Neither

Public Health Impact of long-term, low-level, mixed element exposure in susceptible population strata (PHIME) [43]	**High**	18 mo	2	Neither

Fish Oil and Probiotics in Pregnancy (FOPP) [26]	**Some concerns**	2 y	2	Unclear

Unnamed cohort(s) in Italy [30, 52]	**High/very high**	18–25 mo	4	Unclear

Mixed expressive receptive language	Norwegian Mother and Child Cohort Study (MoBa) [45]	**High**	5 y	5	Favors higher seafood∗	No conclusion
	Mommy's Food Study^3^ [23]	**Some concerns**	11 mo	1	Neither	

	Public Health Impact of long-term, low-level, mixed element exposure in susceptible population strata (PHIME) [24, 43]	**High**	18 mo	3	Neither	
	Nutrition Cohort 1 of the Seychelles Child Development Study^2^ [7]	**Some concerns**	5 y	2	Favors lower seafood	

Fish Oil and Probiotics in Pregnancy (FOPP) [26]	**Some concerns**	2 y	2	Unclear

Two analysts independently reviewed data from each article considering the direction, magnitude, and statistical significance of the reported results, and concluded whether the results indicated that there was either *1*) a relationship between higher seafood intake and better neurocognitive development (“favors higher seafood”), *2*) a relationship between higher seafood intake and worse neurocognitive development (“favors lower seafood”), *3*) no relationship between seafood intake and neurocognitive development (“neither”), or *4*) reported relationships were inconsistent (“inconsistent”). “Unclear” indicates that the data were not reported to determine direction and magnitude of effects or associations. Using the study-level conclusions, each analyst then drafted a suboutcome-level conclusion, and finally, outcome-level conclusions. Discrepancies were resolved through discussion.

∗ Indicates that ≥1 of the results within that suboutcome for that study was statistically significant.

1 All studies are prospective cohort studies unless otherwise indicated.

2 Presumably is Nutrition Cohort 1 based on recruitment date, location, and study and participant characteristics.

3 Randomized controlled trial.

### ADHD

There were 3 PCSs [7,21,50] that assessed relationships between seafood intake during pregnancy and ADHD diagnosis (*n =* 1 study [50]) or traits (*n =* 2 studies [7,21]) in children aged 8–9 y (Table 8). The number of studies was limited, the age range assessed was narrow, and 2 of 3 articles were at high risk of bias due to missing data and reporting bias (Supplemental Table 10). For these reasons, the evidence did not support a conclusion between seafood intake during pregnancy and ADHD in the children.

**TABLE 8:** Seafood during pregnancy and attention-deficit/hyperactivity disorder (ADHD) in the children.

Suboutcome	Study name (references)^1^	Overall risk of bias	Age at outcome assessment	Number of assessments	Study conclusion	Suboutcome conclusion	Outcome conclusion
ADHD diagnosis	The New Bedford Cohort [50]	**High**	8 y	1	Favors higher seafood∗	No conclusion	No conclusion
ADHD index	Infancia y Medio Ambiente [21]	**High**	8 y	6	Favors higher seafood∗	No conclusion	
	Nutrition Cohort 1 of the Seychelles Child Development Study^2^ [7]	**Some concerns**	9 y	2	Neither		

Two analysts independently reviewed data from each article considering the direction, magnitude, and statistical significance of the reported results, and concluded whether the results indicated that there was either *1*) a relationship between higher seafood intake and better neurocognitive development (“favors higher seafood”), *2*) a relationship between higher seafood intake and worse neurocognitive development (“favors lower seafood”), *3*) no relationship between seafood intake and neurocognitive development (“neither”), or *4*) reported relationships were inconsistent (“inconsistent”). “Unclear” indicates that the data were not reported to determine direction and magnitude of effects or associations. Using the study-level conclusions, each analyst then drafted a suboutcome-level conclusion, and finally, outcome-level conclusions. Discrepancies were resolved through discussion.

∗ Indicates that ≥1 of the results within that suboutcome for that study was statistically significant.

1 All studies are prospective cohort studies unless otherwise indicated.

2 Presumably is Nutrition Cohort 1 based on recruitment date, location, and study and participant characteristics.

### Autism spectrum disorder

There were 4 PCSs [28,36,39,41] that assessed relationships between seafood intake during pregnancy and ASD diagnosis (*n =* 1 study [36]) or traits (*n =* 4 [28,36,39,41]) in children aged 6 mo–9 y (Table 9). Although 1 study suggested a relationship between higher seafood and less traits of ASD at 5 y old [39], the other studies were inconsistent [28,36] or suggested no difference [41]. Two articles had some concerns for the risk of bias. The other 2 were at high risk of bias due to confounding, exposure measurement, and reporting bias (Supplemental Table 11). For these reasons, the evidence did not support a conclusion about seafood intake during pregnancy and ASD in the children.

**TABLE 9:** Seafood during pregnancy and autism spectrum disorder in the children.

Suboutcome	Study name (references)^1^	Overall risk of bias	Age at outcome assessment	Number of assessments	Study conclusion	Suboutcome conclusion	Outcome conclusion
ASD diagnosis	Avon Longitudinal Study of Parents and Children [36]	**High**	6 mo–11 y	3	Inconsistent	No conclusion	No conclusion
ASD trait(s)	Infancia y Medio Ambiente [39]	**High**	5 y	11	Favors higher seafood∗	No conclusion	
	Generation R [41]	**Some concerns**	6 y	2	Neither		

	Avon Longitudinal Study of Parents and Children [36]	**High**	3–9 y	21	Inconsistent		

Early Autism Risk Longitudinal Investigation (EARLI) and the Health Outcomes and Measures of the Environment (HOME) Study [28]	**Some concerns**	3–8 y	16	Inconsistent

Abbreviation: ASD, autism spectrum disorder.

Two analysts independently reviewed data from each article considering the direction, magnitude, and statistical significance of the reported results, and concluded whether the results indicated that there was either *1*) a relationship between higher seafood intake and better neurocognitive development (“favors higher seafood”), *2*) a relationship between higher seafood intake and worse neurocognitive development (“favors lower seafood”), *3*) no relationship between seafood intake and neurocognitive development (“neither”), or *4*) reported relationships were inconsistent (“inconsistent”). “Unclear” indicates that the data were not reported to determine direction and magnitude of effects or associations. Using the study-level conclusions, each analyst then drafted a suboutcome-level conclusion, and finally, outcome-level conclusions. Discrepancies were resolved through discussion.

∗ Indicates that ≥1 of the results within that suboutcome for that study was statistically significant.

1 All studies are prospective cohort studies unless otherwise indicated.

### Anxiety or depression

No studies were identified in our search for these 2 outcomes.

## Discussion

The results from this systematic review suggested that there may be a relationship between seafood consumption during pregnancy and better social–emotional and behavioral development outcomes in the children aged 0–18 y as well as better aggregate scores of development from infancy to age <4 y. The range of intakes for the higher seafood comparison groups was between 4 and 30 oz/wk, with most studies reporting between 12 and 16 oz/wk which is at or above current recommendations (8–12 oz/wk of low-mercury options) for pregnancy and lactation [8]. The certainty of evidence was very low to moderate for each conclusion, largely due to reliance on observational studies that were at high risk of bias for confounding and missing data over follow-up. We were unable to conclude how seafood affects other neurocognitive development outcomes due to inconsistent results or a limited number of studies for a given outcome, suboutcome, or age group. However, there may be a relationship between seafood intake during pregnancy and some cognitive and language/communication development outcomes, namely better attention, reasoning and problem-solving, verbal intelligence, communication, and expressive language. However, more research across age groups from study designs with less concerns for bias is needed. Overall, our conclusions align with previous research [4,11,58] in that there were no adverse relationships between seafood intake during pregnancy on child neurocognitive development and that consumption within or even above current recommended amounts may potentially offer benefits for some developmental outcomes. We did not identify any articles that assessed seafood intake during lactation, highlighting an important research gap.

Our results suggested that there was a relationship between seafood intake during pregnancy and better social–emotional, behavioral, and aggregate scores of development in the child, with no adverse relationships observed for other neurocognitive outcomes. A common hypothesis is that the high concentrations of omega-3 fatty acids in seafood are responsible for benefits to neurodevelopment. Omega-3 fatty acids constitute ∼35% of brain cell membranes [59,60] and are essential for neuron development, neurotransmitter functioning and regulation, and gene expression [59,60]. Therefore omega-3 fatty acids from seafood could contribute to improved social–emotional, behavior, and other developmental domains through improvements in the function of the prefrontal cortex. An RCT showed that omega-3 fatty acid supplementation improved the function of the dorsolateral prefrontal cortex [61] whereas impairment of this brain area is associated with antisocial and aggressive behavior [62,63]. Additionally, 2 RCTs suggested small beneficial effects of omega-3 fatty acid supplementation as an adjunct treatment for behavioral or attention disorders for children and adults [60,64]. Thus, it is possible that higher omega-3 fatty acid exposure from seafood intake during pregnancy and lactation may contribute to improved prefrontal cortex functions resulting in better social–emotional and behavioral development among children as well as overall aggregate scores of development.

The previous DGAC review concluded that there was moderate certainty in evidence that seafood intake during pregnancy was associated favorably with cognitive development in young children [11]. In our systematic review, the evidence did not support a conclusion for overall cognitive development due to inconsistency in the direction, magnitude, and statistical significance of results within and between suboutcomes assessed. There are several potential reasons for the discrepancy in our conclusions with those of the previous DGAC review. First, the additional 7 articles identified from 2019 to 2024 [7,19,[23], [24], [25], [26],^28^] reported beneficial [7,19,25], detrimental [23], inconsistent [7,24,28], or unclear [26] associations, adding to the inconsistency of findings reported in the literature. Second, we investigated suboutcomes of cognitive development as a source of heterogeneity because these different aspects of human cognition can be regulated by different regions of the prefrontal cortex [65,66] and should be measured using specific tools at certain ages [67]. On the basis of our suboutcome analyses, the evidence suggested that there may be potential beneficial relationships between seafood intake during pregnancy and attention, reasoning and problem-solving, and verbal intelligence in the child. However, conclusions were not made and the evidence was not graded due to limits in the number of studies, the ages assessed, and the high risk of bias. It is important to note that we made no conclusions that suggested an adverse relationship between seafood during pregnancy and any of the cognitive development suboutcomes assessed. This aligns with conclusions by the 2020 DGAC as well as previous literature despite the use of different synthesis approaches and the addition of new studies.

The certainty of evidence was very low to moderate for our conclusions largely due to issues related to study design. The evidence base was all PCSs, except 1 RCT and 1 retrospective cohort. The high risk of bias was the main reason for downgrading the certainty of evidence for all outcomes from PCSs, particularly for social–emotional and behavioral development. Nonrandomized designs such as PCSs are commonly at higher risk for bias compared with RCTs due to challenges in identifying, measuring, and controlling for all relevant confounding factors. Additionally, dietary exposure assessment in observational studies generally relies on self-reported dietary intake, another source of bias [68]. All but 1 article assessed dietary intake using FFQs, none of which were specifically validated to estimate seafood intake. A further complication and source of bias was variation across studies in how seafood was defined and measured. For example, the term “fish” was used to describe exposures in most articles, which sometimes included shellfish. Accurate quantification of intake from FFQs is challenging due to well-known measurement errors of self-reported dietary data [68]. None of the included articles addressed measurement error which can lead to imprecision in risk estimates and increased type II errors. Therefore, it was difficult to accurately quantify the amount of seafood that may be most beneficial. Conducting RCTs with pregnant and lactating populations has practical, ethical, and liability challenges [69]; therefore, we have to rely heavily on observational research. There is a need for high-quality observational research that uses more rigorous dietary assessment tools, such as repeated weighted food diaries or multiple 24-h recalls or complement self-reported dietary intake with objective markers of seafood intake such as methylmercury to improve this body of literature.

This systematic review highlights important knowledge gaps and needs for future research. First, no articles were identified that assessed seafood intake during lactation. Many infants rely on human milk as a sole nutrition source for the first few months of life [70], a critical period of infant growth and development. A recent systematic review suggested that higher maternal mercury exposure measured via blood and hair samples collected during pregnancy or lactation correlated with higher mercury concentrations in human milk (reference in review). This suggests that maternal mercury exposure may transfer into human milk, highlighting the importance of understanding the relationships between seafood intake, a major source of mercury exposure, during lactation and the neurocognitive development of infants. Second, there is a need for empirical research to investigate relationships between seafood intake and cognitive, movement/physical, and language/communication development outcomes and identify whether certain population characteristics may be modifying associations (e.g. baseline selenium status, mercury exposure from other sources, overall diet quality). These types of data were often not reported in articles included in our systematic review, limiting our investigation into these factors. Finally, research is lacking on how paternal nutrition and sperm health impact child development. Addressing these research gaps would provide a balanced understanding of how seafood affects the span of reproductive and developmental health.

We followed the highest standards of systematic review methodology. Our review would be considered high quality based on AMSTAR 2 criteria [14] (Supplemental Appendix 2), followed PRISMA reporting guidelines for transparency (Supplemental Appendix 1), and adopted a well-vetted protocol developed by the DGAC that was used as one resource to inform dietary guidance in the United States and further reviewed and approved by the NASEM committee. The database search was developed by information specialists with extensive training in evidence synthesis methodology. A limitation of the evidence base was the heterogeneity in outcome assessment tools, scoring systems for the tools, as well as reported statistical comparisons and estimands. This resulted in the *post hoc* organizational decision for the synthesis of adding a new category of “aggregate scores of development” to appropriately capture all the relevant extracted data. The high heterogeneity in results precluded our ability to pool data into a meta-analysis, which was the intended analytical approach in our protocol. Therefore, we relied on narrative synthesis based on the direction, magnitude, and statistical significance of results. This followed a similar synthesis method as the previous DGAC report. However, there are concerns of subjectivity in narrative syntheses, particularly in defining a clinically or biologically meaningful magnitude or effect size for child development outcomes. To alleviate these concerns, we had 2 analysts conduct the narrative syntheses independently and we transparently documented the decisions made at the study-, suboutcome-, and outcome levels.

In this systematic review of mainly PCSs, the evidence suggested that there may be a relationship between seafood consumption and better social–emotional and behavioral development outcomes in children and adolescents aged 0–18 y as well as better aggregate scores of development for those <4 y. Although a conclusion could not be drawn on cognitive development generally, seafood intake during pregnancy may potentially be related to better cognitive-related outcomes of attention, reasoning and problem-solving, and verbal intelligence outcomes. Evidence on ADHD, ASD, and language/communication- and movement/physical-related neurocognitive development was inconclusive.

## Acknowledgments

We acknowledge Darcy Gungor for technical assistance, Cassi N. Uffelman, Rachel C. Thoerig, and Rupal Trivedi for editorial assistance, and Erum Waheed, Harrison Chiu, Poornima Iyer, and Megan Bullard for screening articles (Texas A&M Agriculture, Food and Nutrition Evidence Center; paid contributions).

## Author contributions

The authors’ responsibilities were as follows – MKS, AJM: designed the research and primary responsibility for final content; MKS, LEO, SS, AAB: conducted the research and prepared the data; LEO: analyzed data, wrote the paper with editorial assistance from MKS, AJM, SS, and AAB; and all authors: read and approved the final manuscript.

## Data availability

Data described in the manuscript, code book, and analytic code will be made publicly and freely available without restriction as the Supplemental Data Appendix.

## Funding

This work was supported by a contract with the National Academies of Sciences, Engineering, and Medicine (NASEM). The expert committee convened by NASEM contributed to the search and protocol development.

## Conflict of interest

AJM reports financial support was provided by the National Academies of Sciences, Engineering, and Medicine. LEO reports a relationship with Beef Checkoff that includes: funding grants. LEO, as a previous employee of the USDA and the NIH, has previous and ongoing projects funded by the Beef Checkoff, National Cancer Institute, and National Institute for Food and Agriculture for research unrelated to this work. AJM and MKS were consultants for the National Academies of Sciences, Engineering, and Medicine Committee on the Role of Seafood in Child Growth and Development. If there are other authors, they declare that they have no known competing financial interests or personal relationships that could have appeared to influence the work reported in this paper.
